# Regulatory *nodD1* and *nodD2* genes of *Rhizobium tropici* strain CIAT 899 and their roles in the early stages of molecular signaling and host-legume nodulation

**DOI:** 10.1186/s12864-015-1458-8

**Published:** 2015-03-28

**Authors:** Pablo del Cerro, Amanda Alves Paiva Rolla-Santos, Douglas Fabiano Gomes, Bettina Berquó Marks, Francisco Pérez-Montaño, Miguel Ángel Rodríguez-Carvajal, André Shigueyoshi Nakatani, Antonio Gil-Serrano, Manuel Megías, Francisco Javier Ollero, Mariangela Hungria

**Affiliations:** Departamento de Microbiología, Facultad de Biología, Universidad de Sevilla, Avda. Reina Mercedes, 6 Apdo Postal 41012 Sevilla, Spain; Embrapa Soja, C.P. 231, 86001-970 Londrina, Paraná Brazil; Departamento de Química Orgánica, Facultad de Química, Universidad de Sevilla, Apdo Postal 553, 41071 Sevilla, Spain

**Keywords:** *nodD* gene, Nod factors, Nodulation, Symbiosis, Nitrogen fixation, *Rhizobium tropici*

## Abstract

**Background:**

Nodulation and symbiotic nitrogen fixation are mediated by several genes, both of the host legume and of the bacterium. The rhizobial regulatory *nodD* gene plays a critical role, orchestrating the transcription of the other nodulation genes. *Rhizobium tropici* strain CIAT 899 is an effective symbiont of several legumes—with an emphasis on common bean (*Phaseolus vulgaris*)—and is unusual in carrying multiple copies of *nodD*, the roles of which remain to be elucidated.

**Results:**

Phenotypes, Nod factors and gene expression of *nodD1* and *nodD2* mutants of CIAT 899 were compared with those of the wild type strain, both in the presence and in the absence of the *nod*-gene-inducing molecules apigenin and salt (NaCl). Differences between the wild type and mutants were observed in swimming motility and IAA (indole acetic acid) synthesis. In the presence of both apigenin and salt, large numbers of Nod factors were detected in CIAT 899, with fewer detected in the mutants. *nodC* expression was lower in both mutants; differences in *nodD1* and *nodD2* expression were observed between the wild type and the mutants, with variation according to the inducing molecule, and with a major role of apigenin with *nodD1* and of salt with *nodD2*. In the *nodD1* mutant, nodulation was markedly reduced in common bean and abolished in leucaena (*Leucaena leucocephala*) and siratro (*Macroptilium atropurpureum*), whereas a mutation in *nodD2* reduced nodulation in common bean, but not in the other two legumes.

**Conclusion:**

Our proposed model considers that full nodulation of common bean by *R. tropici* requires both *nodD1* and *nodD2,* whereas, in other legume species that might represent the original host, *nodD1* plays the major role. In general, *nodD2* is an activator of *nod*-gene transcription, but, in specific conditions, it can slightly repress *nodD1. nodD1* and *nodD2* play other roles beyond nodulation, such as swimming motility and IAA synthesis.

**Electronic supplementary material:**

The online version of this article (doi:10.1186/s12864-015-1458-8) contains supplementary material, which is available to authorized users.

## Background

Bacteria commonly known as rhizobia are capable of establishing symbioses with several leguminous species, forming specific structures, the root nodules, where the process of biological fixation of atmospheric nitrogen takes place, bringing important contributions to agriculture and to the environment [1-3]. Legume nodulation requires a cascade of molecular signals exchanged between the host plant and the rhizobium. This molecular dialogue begins with the exudation of flavonoids from the legume, which are recognized by the bacterium. When induced by these plant molecules, rhizobia synthesize lipochitooligosaccharides (LCOs), also known as Nod factors, responsible for launching the nodulation process [3-8]. It is noteworthy that an increasing number of reports show that Nod factors may play roles beyond the nodulation process, including stimulation of photosynthesis, improvements in plant growth and grain yield and changes in immune responses in both legumes and non-legumes [9-12].

We consider *nodD* as the most intriguing regulatory nodulation gene; it belongs to the LysR family of transcriptor regulators, and it is constitutively expressed and responsible for the transcription of other nodulation genes in the presence of suitable plant inducers, usually flavonoids, thus initiating the nodulation process [8,13,14]. Furthermore, many other symbiosis-related phenotypes, such as polysaccharide production, phytohormone synthesis, motility, quorum-sensing and the activation of the type-III secretion system are directly or indirectly regulated by means of inducing flavonoids via NodD in rhizobia [15-20]. Studies of genomes of rhizobia indicate that, depending on the rhizobial species, there are one to five copies of *nodD*. In the species that possess only one copy of this gene, such as *Rhizobium leguminosarum* bv. trifolii, a mutation usually results in loss of nodulation, whereas, in the presence of multiple copies, as in *Sinorhizobium* (=*Ensifer*) *meliloti, Rhizobium leguminosarum* bv. phaseoli and *Bradyrhizobium japonicum,* an intricate interaction between the *nodD* genes seems to occur and the nodulation is not completely suppressed [21-23].

*Rhizobiun tropici* strain CIAT 899 is an effective microsymbiont of common bean (*Phaseolus vulgaris* L.) in the tropical acid soils of South America. Notable properties of this strain are its high tolerance of environmental stresses and its broad legume host-range [24-26]. Another intriguing feature of CIAT 899 is its capacity for producing a large variety of Nod factors [27,28]. Interestingly, this bacterium is able to produce these key symbiotic molecules under abiotic stresses, such as acid and saline conditions, in the absence of plant-molecular signals [28-30]. In a pioneering study, five distinct *nodD*-hybridizing DNA regions were identified in CIAT 899 [31], later confirmed as five *nodD* genes in the sequenced genome of the strain [32]. The *nodD1* gene preceding the *nodABC* operon seems to play the major role in nodulation [31], but a more precise study of the regulatory functions of *nodD1* in *R. tropici* is lacking. In addition, the role of *nodD2*, present in some rhizobial species, is unclear. It has been reported to be a repressor of the *nodABC* operon, leading to a negative effect on Nod-factor production in *Sinorhizobium* (=*Ensifer*) *fredii* strain NGR 234 [33]. A suppressive role has also been observed in *B. japonicum* [23] and a negative regulation by NodD2 products was reported in *Bradyrhizobium* (*Arachis*) [34].

Here we report a study in which phenotypes, Nod factors and gene expression of mutants of nodulation genes *nodD1* and *nodD2* of strain CIAT 899 were compared with those of the wild type strain, to help to elucidate the roles of these regulatory genes.

## Results and discussion

### Phenotypic characterization of wild type and mutant strains

As defined in the genome of *R. tropici* strain CIAT 899 [32], *nodD1* precedes the *nodABC* operon, while *nodD2*, corresponding to *nodD5* described by van Rhijn *et al.* [31], is adjacent to the *nodA2* and *hsnT* genes (Additional file 1: Figure S1). *R. tropici* CIAT 899 *nodD1* mutant was obtained in a previous work by insertion of a Km^R^ cassette into a unique *Xho*I restriction site located on the gene [30]. As described in the Material and Methods section, the *nodD2* mutant was obtained after deletion of a 0.6 kb *Ps*tI fragment of the gene and the insertion in its place of the Ω interposon (Additional file 1: Figure S1).

**Figure 1:**
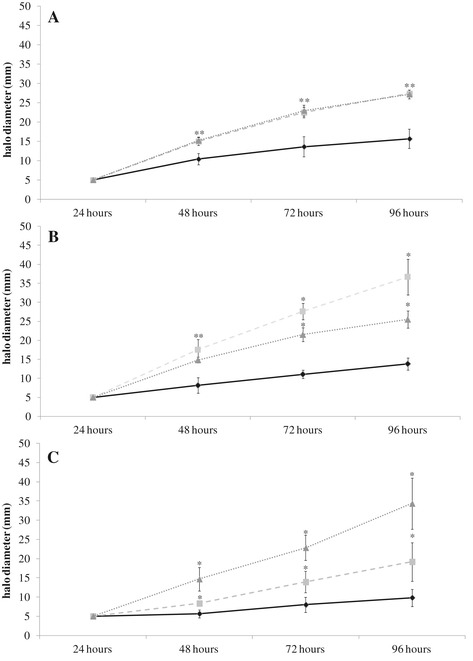
**Swarming motility phenotype of the** ***R. tropici*** **CIAT 899 wild type and** ***nodD1*** **and** ***nodD2*** **mutants.** Quantified swarm ring diameters of wild type strain (continuous line), the *nodD1* mutant (striped line) and the *nodD2* mutant (dotted line). Values are the averages of three swarm plates per strain. *nodD1* and *nodD2* mutant parameters were individually compared with the parental strain CIAT 899 parameters by using the Mann–Whitney non-parametric test. Values tagged by * are significantly different at the level α = 5%. Swarming motility in: **A**. TY medium, **B**. TY medium supplemented with 3.7 μM of apigenin, and **C**. TY medium supplemented with 300 mM of NaCl.

**Figure 2:**
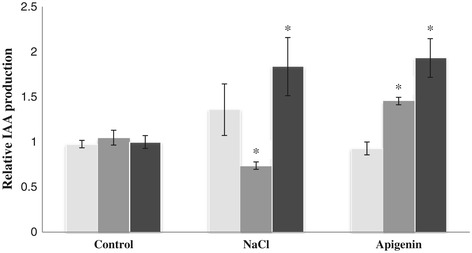
**Indole-3-acetic acid (IAA) relative production by** ***R. tropici*** **CIAT 899 wild type, and by the** ***nodD1*** **and** ***nodD2*** **mutants.** Bacteria were grown in TY medium containing tryptophan in absence and presence of apigenin (3.7 μM) or NaCl (300 mM). Supernatants were taken 96 h after the addition of flavonoid or salt. IAA production was calculated relative to the production without inducing molecules of the wild type strain by using the Mann–Whitney non-parametrical test. The asterisks indicate a significant different at the level α = 5%. Black bars: CIAT 899. Light gray bars: *nodD1* mutant. Dark gray bars: *nodD2* mutant.

Growth rate was not affected by mutation in *nodD1* or *nodD2* genes of *R. tropici* CIAT 899 (data not shown). However, it is known that some bacterial properties may be regulated via NodD proteins, such as EPS (exopolysacharide) production, LPS (lipopolysaccharide) profiles, swimming and swarming motilities, biofilm formation and IAA (indole acetic acid) synthesis, among others (e.g., [15-20]). We evaluated some these properties in the wild type and mutant strains in the presence or absence of two *nod*-gene inducing molecules, apigenin (3.7 μM) and salt (NaCl 300 mM). Results showed statistical differences only in swarming motility (Figure 1) and in the production of IAA (Figure 2).

Swarming motility is caused by rotation of single or multiple flagellae along wetted surfaces [35] and, in certain rhizobia, is altered in the presence of legume root exudates that are rich in flavonoids [36]. Our experiments showed that, under control conditions, both *nodD* mutants showed more swarming motility than did the wild type strain. Interestingly, these differences were even stronger when the medium was augmented with apigenin for the *nodD1* mutant and with NaCl for the *nodD2* mutant (Figure 1). Therefore, the results suggest a constitutive suppression of swarming by NodD1 and NodD2 proteins.

IAA is an essential plant hormone that promotes growth, including lateral-root proliferation. Previous work has demonstrated that synthesis of this molecule is regulated by NodD1 and NodD2 in *S. fredii* strain NGR234 [16]. In CIAT 899, our experiments showed an increase in the production of IAA in the presence of apigenin and NaCl (Figure 2), suggesting that both inducing molecules promote the synthesis of this phytohormone. This finding is supported by the presence of a *nod*-box upstream of the IAA operon in the genome of CIAT 899 [32]. In addition, NodD1 seems to be the main regulator in the presence of apigenin, since, in this mutant, the production of IAA was significantly lower than in the presence of NaCl. The production of IAA in the *nodD2* mutant was strongly reduced when the medium was supplemented with NaCl (Figure 2), suggesting that this regulator may be mainly implied in the activation of the IAA operon in the presence of salt. Altogether, the results indicate a predominant role of NodD1 in activation of the IAA gene by apigenin and a predominant role of NodD2 when the inducing molecule is NaCl.

The nodulation phenotype in common bean was first evaluated in pouches bags, where it was possible to observe that a mutation in *nodD1* caused a significant decrease in nodule number of common bean; to a lesser extent, a decrease was also observed with a mutation in *nodD2* (Additional file 2: Figure S2). In both leucaena [*Leucaena leucocephala* (Lam.) de Wit] and siratro [*Macroptilium atropurpureum* (DC.) Urb.], no nodules were observed when plants were inoculated with the *nodD1* mutant, but apparently no differences were observed when plants of both species were inoculated with the *nodD2* mutant in comparison with plants inoculated with the wild type strain (data not shown).

Nodulation of the type and mutants was confirmed by growing plants in larger pots, in Leonard jars containing sterile substrate. In common bean, a mutation in *nodD1* did not suppress nodulation, but caused a reduction of 82% in nodule number (Table 1). The absence of nodulation in both leucaena and siratro when inoculated with the *nodD1* mutant was confirmed. *Vis-à-vis* the *nodD2* mutant, nodulation of common bean was reduced by 55%, and no statistical differences in relation to the wild type strain were observed in the nodulation of either leucaena or siratro. However, shoot dry weight of the leucaena plants inoculated with the *nodD2* mutant was lower than with the wild type (α = 10%) (Table 1).

**Table 1:** **Nodule number (n° plant** ^**−1**^ **) and shoot dry weight (g plant** ^**−1**^ **) of common bean, leucaena and siratro inoculated with** ***R. tropici*** **strain CIAT 899 and** ***nodD*** **derivatives**

**Strains**	***P. vulgaris*** ^**a**^		***L. leucocephala*** ^**a**^		***M. atropurpureum*** ^**a**^	
	**Nodule number**	**Shoot dry weight**	**Nodule number**	**Shoot dry weight**	**Nodule number**	**Shoot dry weight**
*R. tropici* CIAT899	213 ± 52	1.82 ± 0.64	13 ± 4	0.41 ± 0.03	34 ± 8	0.05 ± 0.01
*nodD1* mutant	38 ± 11*	1.42 ± 0.35	0 ± 0*	0.09 ± 0.01*	0 ± 0*	0.05 ± 0
*nodD2*mutant	95 ± 38*	1.03 ± 0.27	10 ± 3	0.36 ± 0.04**	24 ± 8	0.05 ± 0
none	0 ± 0*	0.80 ± 0.25*	0 ± 0*	0.09 ± 0.01*	0 ± 0*	0.05 ± 0

^a^Data represent means ± SD (standard deviation) of six jars, each with two plants. *nodD1* and *nodD2* mutant parameters were individually compared with the parental strain CIAT 899 parameters by using the Mann–Whitney non-parametric test. Values tagged by *and **are significantly different at the level α = 5 and 10%, respectively.

Plants evaluated after 25 (common bean) or 42 days (leucaena and siratro) of growth under controlled conditions.

In rhizobial species with more than one copy of the regulatory *nodD* gene, *nodD1* preceding the *nodABC* operon has been recognized as the main gene regulating nodulation *e.g.* [23,33,34,37-39]. However, reports show that the role of each *nodD* copy, their responses to flavonoids, and the nodulation phenotypes vary on a case-by-case basis with the rhizobium strain and the host-plant species/cultivar. An intricate pattern of responses in nodulation leads to the assumption that *S. meliloti* utilizes the three copies of *nodD* to optimize the interaction with each of its legume hosts [37,40]. A mutation in *nodD1* of *S. meliloti* delays but does not eliminate nodulation of both alfalfa (*Medicago sativa*) and sweet clover (*Melilotus alba*), and only a triple mutation of *nodD1*-*nodD2*-*nodD3* results in absence of nodules [37]. Contrarily, in the promiscuous strain *S. fredii* NGR 234, capable of nodulating more than 110 plant species, a mutation in *nodD1* abolishes nodulation in several temperate and tropical species [41]. In addition, in *B. japonicum nodD1* is sufficient for nodulation of the putative main host plant, soybean (*Glycine max*), but the additional genes *nodVW* are required for the nodulation of mung bean (*Vigna radiata*), cowpea (*Vigna unguiculata*) and siratro [39,42]. In our study, a mutation in *nodD1* decreased, but did not suppress, nodulation of common bean; however, *nodD1* proved to be essential for the nodulation both of leucaena and of siratro (Table 1).

Still considering nodulation phenotype, in *S. meliloti* the *nodD2* gene did not have any apparent effect on nodulation of either alfalfa or sweet clover [37]. Similarly, no detectable effects were observed by inoculating siratro and cowpea with a *nodD2* mutant of *Bradyrhizobium* (*Arachis*) sp. strain NC92 [34]. Contrarily, in our study, a significant decrease in nodulation of common bean was detected with the mutation in *nodD2*, but no effects were observed in leucaena and siratro (Table 1).

### Nod factor patterns

*Rhizobium tropici* strain CIAT 899 is known as an interesting strain in relation to its production of a large variety of Nod factors, not only when induced by flavonoids [27,28], but also under high-salinity conditions in the absence of flavonoids [28-30].

A list of all Nod factors detected in the wild type strain in comparison to the *nodD1* and *nodD2* mutants is shown in Tables 2, 3 and 4. Unexpectedly, Nod factors were found in the B^−^ medium [43], even in the absence of inducer molecules. In this condition, around ten Nod factors were synthesized, with no significant differences among wild type CIAT 899, *nodD1* and *nodD2* mutants (Table 2). When induced by 3.7 μM apigenin, the synthesis of a variety of Nod factors was confirmed in all strains, such that numerically, 29 Nod factors were detected in the wild type CIAT 899 and 25 in the *nodD2* mutant; a slight reduction was observed with the *nodD1* mutant, but, even then, 20 Nod factors were observed (Table 3). This number is higher than in other wild type rhizobial species, *e.g.*the four Nod factors identified in *B. japonicum* strain USDA 138 [44]. Up to 36 Nod factors were found in CIAT 899 under saline conditions (Table 4), and in the *nodD1* and *nodD2* mutants the numbers were lower, of 20 and 18 Nod factors, respectively. These results indicate that NaCl has a stronger *nod*-induction capacity than apigenin does, and that it is affected by *nodD2* but not *nodD1*. However, one might also consider that it deserves further studies to investigate the possibility that Nod factors are more stable in a 300 mM NaCl supplemented medium.

**Table 2:** **Nod factor structure biosynthesized in control condition (B** ^**−**^ **medium) by wild type CIAT 899 and the** ***nodD1*** **and** ***nodD2*** **mutants**

**[M + H]** ^**+**^ **(** ***m*** **/** ***z*** **)**	**B**_**i**_ **ions**	**Structure** ^**a**^	**CIAT899** ^**b**^	***nodD1*** ^**b**^	***nodD2*** ^***b***^
850	426, 629, 832	III (C_18:1_)	+	+	-
1027	400, 603, 806	IV (C_16:0_)	+	+	+
1041	414, 617, 820	IV (C_16:0_, NMe)	-	-	+
1053	426, 629, 832	IV (C_18:1_)	+	+	+
1055	428, 631, 834	IV (C_18:0_)	+	-	-
1067	440, 643, 846	IV (C_18:1_, NMe)	+	+	+
1216	386, 589, 792, 995	V (C_14:0_, NMe)	+	-	+
1230	400, 603, 806, 1009	V (C_16:0_)	+	+	+
1244	414, 617, 820, 1023	V (C_16:0_, NMe)	+	+	+
1256	426, 629, 832, 1035	V (C_18:1_)	+	+	+
1270	440, 643, 846, 1049	V (C_18:1_, NMe)	+	+	+
1350	440, 643, 846, 1049, [M-80]^+c^ = 1270	V (C_18:1_, NMe, S)	+	+	+
1352	442, 645, 848, 1051, [M-80]^+c^ = 1272	V(C_18:0_, NMe, S)	-	+	-
1378	468, 671, 874, 1077, [M-80]^+c^ = 1298	V (C_20:1_, NMe, S)	-	+	-

^a^NF structures are represented following the convention (Spaink, 1992) [43] that indicates the number of GlcNAc residues in the backbone (Roman numeral), the length and degree of unsaturation of the fatty acyl chain, and the other substituents, which are listed in the order in which they appear, moving clockwise from the fatty acid. NMe, *N*-methyl group at glucosamine non reducing residue; S, sulfate group at reducing glucosamine residue.

^b^Symbol: + = detected; − = non detected.

^c^These ions arise by loss of a neutral with mass 80 Da, corresponding to the loss of SO_3_.

**Table 3:** **Nod factor structure biosynthesized in the presence of apigenin (3.7** μ**M) by wild type CIAT899 and the** ***nodD1*** **and** ***nodD2*** **mutants**

**[M + H]** ^**+**^ **(** ***m*** **/** ***z*** **)**	**B**_**i**_ **ions**	**Structure** ^**a**^	**CIAT899** ^**b**^	***nodD1*** ^**b**^	***nodD2*** ^**b**^
**810**	386, 589	III (C_14:0_, NMe)	-	-	+
**824**	400, 603	III (C_16:0_)	-	+	+
**838**	414, 617	III (C_16:0_, NMe)	+	+	+
**850**	426, 629	III (C_18:1_)	+	+	+
**852**	428, 631	III (C_18:0_)	+	-	-
**864**	440, 643	III (C_18:1_, NMe)	+	-	+
**999**	372, 575, 778	IV (C_14:0_)	+	+	-
**1011**	384, 597, 790	IV (C_14:1_, NMe)	+	-	-
**1013**	386, 589, 792	IV (C_14:0_, NMe)	+	+	+
**1025**	398, 601, 804	IV (C_16:1_)	+	+	+
**1027**	400, 603, 806	IV (C_16:0_)	+	+	+
**1039**	412, 615, 818	IV (C_16:1_, NMe)	+	+	+
**1041**	414, 617, 820	IV (C_16:0_, NMe)	+	+	+
**1053**	426, 629, 832	IV (C_18:1_)	+	+	+
**1055**	428, 631, 834	IV (C_18:0_)	+	+	+
**1067**	440, 643, 846	IV (C_18:1_, NMe)	+	-	+
**1069**	442, 645, 848	IV (C_18:0_, NMe)	+	-	+
**1081**	454, 657, 860	IV (C_20:1_)	+	-	-
**1147**	440, 643, 846	IV (C_18:1_, NMe, S)	-	-	+
**1202**	372, 575, 778, 981	V (C_14:0_)	+	+	-
**1214**	426, 629, 790, 832, 993^d^	V (C_18:1_, dNAc)	+	-	-
**1216**	386, 589, 792, 995	V (C_14:0_, NMe)	+	+	+
**1228**	440, 643, 846, 1007^e^	V (C_18:1_, NMe, dNAc)	+	-	-
**1230**	400, 603, 806, 1009	V (C_16:0_)	+	+	-
**1231**	440, 643, 846, 1049	IV Hex-ol (C_18:1_, NMe)	-	-	+
**1242**	412, 615, 818, 1021	V (C_16:1_, NMe)	+	+	+
**1244**	414, 617, 820, 1023	V (C_16:0_, NMe)	+	+	+
**1256**	426, 629, 832, 1035	V (C_18:1_)	+	+	+
**1270**	440, 643, 846, 1049	V (C_18:1_, NMe)	+	+	+
**1272**	442, 645, 848, 1051	V (C_18:0_, NMe)	+	-	+
**1284**	454, 657, 860, 1063	V (C_20:1_)	-	-	+
**1324**	414, 617, 820, 1023	V (C_16:0_, NMe, S)	+	-	+
**1336**	426, 629, 832, 1035	V (C_18:1_, S)	+	+	-
**1350**	440, 643, 846, 1049, [M-80]^+c^ = 1270	V (C_18:1_, NMe, S)	+	+	+

^a^NF structures are represented following the convention (Spaink, 1992) [43] that indicates the number of GlcNAc residues in the backbone (Roman numeral), the length and degree of unsaturation of the fatty acyl chain, and the other substituents, which are listed in the order in which they appear, moving clockwise from the fatty acid. Hex-ol, hexytol (reduced terminal hexose); NMe, *N*-methyl group at glucosamine non reducing residue; S, sulfate group at reducing glucosamine residue.

^b^Symbol: + = detected; − = non detected.

^c^These ions arise by loss of a neutral with mass 80 Da, corresponding to the loss of SO_3_.

^d^Mixture of two Nod Factors, deacetylated at glucosamine residues numbers 2 and 3, respectively.

^e^Nod Factor deacetylated at glucosamine residue number 2.

**Table 4:** **Nod Factor structure biosynthesized in the presence of 300 mM NaCl by wild type CIAT899 and the** ***nodD1*** **and** ***nodD2*** **mutants**

**[M + H]** ^**+**^ **(** ***m*** **/** ***z*** **)**	**B**_**i**_ **ions**	**Structure** ^**a**^	**CIAT 899** ^**b**^	***nodD1*** ^**b**^	***nodD2*** ^**b**^
824	400, 603	III (C_16:0_)	+	+	+
838	414, 617	III (C_16:0_, NMe)	+	+	+
850	426, 629	III (C_18:1_)	+	+	+
864	440, 643	III (C_18:1_, NMe)	+	-	+
999	372, 575, 778	IV (C_14:0_)	+	+	-
1013	386, 589, 792	IV (C_14:0_, NMe)	+	+	-
1025	398, 601, 804	IV (C_16:1_)	+	+	+
1027	400, 603, 806	IV (C_16:0_)	+	+	+
1041	414, 617, 820	IV (C_16:0_, NMe)	+	+	+
1053	426, 629, 832	IV (C_18:1_)	+	+	+
1055	428, 631, 834	IV (C_18:0_)	+	-	-
1067	440, 643, 846	IV (C_18:1_, NMe)	+	+	+
1069	442, 645, 848	IV (C_18:0_, NMe)	+	-	-
1147	440, 643, 846	IV (C_18:1_, NMe, S)	+	-	-
1149	442, 645, 848	IV (C_18:0_,NMe, S)	+	-	-
1202	372, 575, 778, 981	V (C_14:0_)	-	+	-
1203	414, 617, 820, 1023	IV Hex (C_16:0_, NMe)	+	-	-
1205	414, 617, 820, 1023	IV Hex-ol (C_16:0_, NMe)	+	-	-
1215	426, 629, 832, 1035	IV Hex (C_18:1_)	+	+	-
1216	386, 589, 792, 995	V (C_14:0_, NMe)	+	+	+
1229	440, 643, 846, 1049	IV Hex (C_18:1_, NMe)	+	-	-
1230	400, 603, 806, 1009	V (C_16:0_)	+	+	+
1231	440, 643, 846, 1049	IV Hex-ol (C_18:1_, NMe)	+	-	-
1233	442, 645, 848, 1051	IV Hex-ol (C_18:0_, NMe)	+	-	-
1242	412, 615, 818, 1021	V (C_16:1_, NMe)	+	+	+
1244	414, 617, 820, 1023	V (C_16:0_, NMe)	+	+	+
1256	426, 629, 832, 1035	V (C_18:1_)	+	+	+
1258	428, 631, 834, 1037	V (C_18:0_)	+	-	-
1270	440, 643, 846, 1049	V (C_18:1_, NMe)	+	+	+
1272	442, 645, 848, 1051	V (C_18:0_, NMe)	+	-	-
1298	468, 671, 874, 1077	V (C_20:1_, NMe)	+	-	-
1324	414, 617, 820, 1023	V (C_16:0_, NMe, S)	+	-	+
1336	426, 629, 832, 1035	V (C_18:1_, S)	+	+	+
1350	440, 643, 846, 1049, [M-80]^+c^ = 1270	V (C_18:1_, NMe, S)	+	+	+
1352	442, 645, 848, 1051	V (C_18:0_, NMe, S)	+	-	-
1378	468, 671, 874, 1077	V (C_20:1_, NMe, S)	+	-	-
1380	470, 673, 876, 1079	V (C_20:0_, NMe, S)	+	-	-

^a^NF structures are represented following the convention (Spaink, 1992) [43] that indicates the number of GlcNAc residues in the backbone (Roman numeral), the length and degree of unsaturation of the fatty acyl chain, and the other substituents, which are listed in the orr in which they appear, moving clockwise from the fatty acid. Hex, hexose; Hex-ol, hexytol (reduced terminal hexose); NMe, *N*-methyl group at glucosamine non reducing residue; S, sulfate group at reducing glucosamine residue.

^b^Symbol: + = detected; − = non detected.

^c^These ions arise by loss of a neutral with mass 80 Da, corresponding to the loss of SO_3_.

The production of a large number of Nod factors in all conditions tested might be related to broad host promiscuity and abiotic-stress tolerance of *R. tropici* [24-26]. The promiscuous *S. fredii* strain NGR 234 also produces a larger number of Nod factors (≥18) [41], and the composition of Nod factors produced by this strain varies with the activity of host-specific nodulation genes [45]. Furthermore, one interesting feature observed in our study was that the Nod factors with structure III (C_18:1_, NMe), IV (C_18:1_, NMe), IV (C_18:0_, NMe), V (C_18:0_, NMe) and V (C_16:0_, NMe, S) were present in the wild type and in the *nodD2* mutant, but not in the *nodD1* mutant; therefore, this structure might be implicated in host-specific nodulation, and could explain why the mutant in the *nodD1* gene is unable to induce nodules on leucaena or siratro. It is also worth mentioning that Folch-Mallol *et al.* [46] described that in CIAT 899 the sulfation of the LCOs, mediated by the *nodHPQ* genes are important for nodulation efficiency on *L. leucocephala*. A mutant in the *nodH* gene induced about half of nodules than those induced by the wild type strain [46]. Interestingly, one of the five LCOs not synthesized by the CIAT 899 *nodD1* mutant is sulphated [V (C16: 0, NMe, S)] (Tables 3 and 4) and may be important for nodulation on leucaena*.* However, because the *nodD1* mutant is unable to nodulate leucaena, other LCOs not secreted by this mutant must be important to explain its symbiotic phenotype.

In *R. tropici*, the amount and diversity of Nod factors produced are directly influenced by the conditions of bacterial growth. Our results are consistent with the report that CIAT 899 produces of a high number of Nod factors in the presence of *nod*-gene-inducing molecules [27-30], which provides a better understanding of the control of Nod-factor biosynthesis, and which, in *R. tropici*, does not follow the classical pathway mediated by flavonoids.

### Gene expression

**Figure 3:**
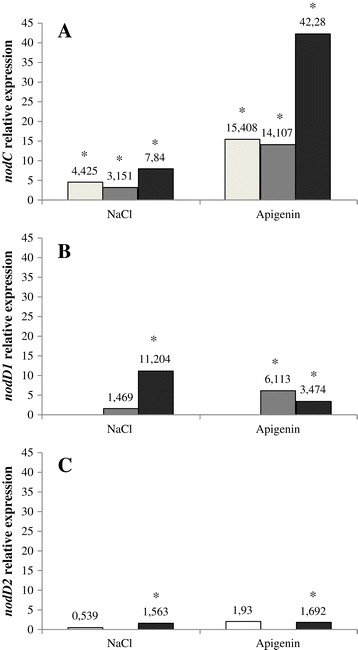
**RT-qPCR analysis of the expression of several** ***nod*** **genes from wild type** ***R. tropici*** **CIAT 899 and** ***nodD1*** **and** ***nodD2*** **mutants grown in absence and presence of apigenin (3.7** **μM) or NaCl (300 mM).** Expression data shown are the mean of three biological replicates. Data were normalized in relation to the endogenous control (16S rRNA). The asterisks indicate a statistically significant expression at the level α = 5%, determined by REST2009 software. Light gray bars: *nodD1* mutant, dark gray bars: *nodD2* mutant, black bars: wild type strain. **A**. *nodC* expression. **B**. *nodD1* expression. **C**. *nodD2* expression.

In various strains of rhizobia, the *nodD1* gene is the chief regulator of Nod-factor biosynthesis and symbiotic phenotype *e.g.* [34,37,38,47]. Contrarily, *nodD2* has been proposed as a repressor of *nod*-gene expression [33,39,48,49], affecting the bacterial Nod-factor profile. We performed gene expression studies with the wild type and *nodD1* and *nodD2* mutants, to improve our understanding of the roles of these two genes (Figure 3).

We evaluated the relative expression of the *nodC* gene (Figure 3A), which controls the elongation of the oligosaccharide chain of Nod factors and is transcribed with the activation of *nod* genes. The relative expression of *nodC* was lower for both mutants in comparison to the wild type strain, both in the apigenin and in the salt treatments (Figure 3A).

Significant expressions of the *nodD1* gene was observed in the WT strain both with salt and apigenin, while for the *nodD2* mutant it was statistically significant only when induced with apigenin, and higher than in the WT (Figure 3B).

In relation to the expression of *nodD2*, CIAT 899 WT strain significantly expressed the gene both with salt and apigenin. Contrarily, no statistically significant expression was observed for the *nodD1* mutant in none of the conditions evaluated (Figure 3C). However, we must consider that the expression levels of *nodD2* were all very low, and numerically even higher for the *nodD1* mutant in the presence of apigenin, therefore no strong conclusion can be taken from this assay at this moment (Figure 3C).

All together, these results indicate that the *nodD1 is a* positive regulator gene, while *nodD2* may positively or negatively regulate the expression of the *nodD1* gene. Supporting these results is evidence of the involvement of *nodD2* in the regulation of the expression of *nodD1* by binding to *nod box*-like sequences located upstream of its coding region [33,49].

### Proposal of a regulatory model for *nodD1* and *nodD2* genes of *R. tropici*

A graphic summary of the main features of the wild type CIAT 899 and *nodD1* and *nodD2* mutants is shown in Additional file 3: Figure S3. In our study a major role of *nodD1* in *R. tropici* CIAT 899 was confirmed. In the presence of the *nod*-gene-inducer apigenin, *nodD1* greatly increased the expression of *nodC* (42-fold), decreasing to 15-fold when the gene was mutated. Similar responses, but lower in magnitude, were observed under saline conditions (Figure 3A). Although confirming a major role of *nodD1*, the results also indicate that other *nodD* genes are involved in the activation of *nodC*, in the presence both of flavonoids and of salts.

Still in relation to *nodD1*, a mutation abolished nodulation in leucaena and siratro, but not in common bean. As *nodD1* gene is the chief regulator of Nod-factor biosynthesis and thus nodulation of the host plant *e.g.* [5,8,43,44], our results suggest that common bean might not be the main host of *R. tropici*, although it has been largely isolated from this host legume in acid soils of South America [24,26,50-52]. Indeed, doubts about common bean as the main host of *R. tropici* have been raised, giving support to the hypothesis that the species might be an original symbiont of another indigenous legume, further “adapting” to common bean [50]. *R. tropici* has been isolated from common bean and other indigenous legumes in Europe, Africa, Australia, and North America [50], and results of some studies suggest the following as original host candidates for *R. tropici*: *Gliricidia* spp., from which the strain has been isolated in Mexico [53] and Brazil [54]; *Acaciella angustissima* in Mexico [55]; and *Mimosa* spp. in Brazil [54]*.*

Understanding the relation between Nod factors and host specificity has been a goal of several studies, but without full success. In our research, we found that Nod factors of the following structures, III (C_18:1_, NMe), IV (C_18:1_, NMe), IV (C_18:0_, NMe),V (C_18:0_, NMe) and V (C_16:0_, NMe, S) (Tables 3 and 4) might be related—to a greater or lesser extent—to the nodulation of the original host plant, as they are absent in the *nodD1* mutant. We have also confirmed the great variety of Nod factors produced by *R. tropici*, as reported before [27-30], even in the absence of *nod*-gene inducers [29,30] (Additional file 3: Figure S3). We propose a new, constitutive mechanism of Nod-factor synthesis that is highly enhanced when environmental conditions are stressful, such as strongly acidic pH or salinity. Some transcriptional regulators may be activated in these conditions and they could be responsible for the regulation of *nod*-gene expression via *nodD* regulators.

In various rhizobial strains, *nodD2* has been described as a repressor of the expression of *nod* genes *e.g.* [23,33,39,49]. For example, in *B. japonicum*, induction of *nodC* by flavonoids is virtually suppressed by elevated levels of NodD2 [23], and in *S. fredii* extra plasmid copies of *nodD2* reduced the level of *nodD1* transcripts to below the limits of detection [49]. However, there is still no evidence that the suppression by NodD2 is mediated by *nodD1*. In our study, we found that *nodD2* activated *nodC* at similar levels as those observed for *nodD1* with both apigenin and salt. However, a slight repression of *nodD1* by *nodD2* was observed in the presence of apigenin (Figure 3B). Accordingly, we hypothesize that *nodD2* is usually an activator of *nod*-gene transcription, although, in the presence of some flavonoids it may slightly repress *nodD1*. Nevertheless, if this repression is biologically significant, it remains to be determined, as no differences in nodulation were observed for leucaena or siratro in the absence of *nodD2*, whereas nodulation was decreased in common bean.

Our model contends that full nodulation of common bean by *R. tropici* requires both *nodD1* and *nodD2,* while, in other plant species that might represent the original host, *nodD1* plays the major role. *nodD2* is not a strong repressor as described in other rhizobial species, and, in general, plays a role as an activator of *nod*-gene transcription, but, in specific conditions, it may slightly repress *nodD1*. The *nodD* regulation in *R. tropici* CIAT 899 resembles the pattern observed in *S. meliloti*—need for three copies of *nodD* to optimize the interaction with each of its legume hosts [40]. The biological significance of producing an abundance of Nod factors is not completely understood yet, but we hypothesize that represents an evolutionary strategy to avoid abiotic stresses by nodulating a range of legume species. Reports show that *nod* genes may also control other functions that contribute to nodulation, as described for *nodD2* in the exopolysaccharide synthesis of *S. fredii* [38], and chaperones and other genes by *nodD1* in *S. meliloti* [56], *inter alia*. Our results demonstrate extra roles for *nodD1* and *nodD2* of *R. tropici* in swarming motility and IAA synthesis.

## Conclusions

Our model proposes that full nodulation of common bean by *R. tropici* requires both *nodD1* and *nodD2,* while in other plant species that might represent the original host *nodD1* plays the major role. Contrarily to other rhizobial species, *nodD2* of *R. tropici* is usually not a strong repressor of *nod*-gene transcription. *R. tropici* synthesizes a variety of Nod factors that might be related to the ability of nodulating a variety of legume species, representing an evolutionary strategy of the symbiosis under abiotic stressful conditions. *nodD1* and *nodD2* of *R. tropici* also play roles in swarming motility and IAA synthesis.

## Methods

### Bacterial strains, plasmids, media, and growth conditions

*Rhizobium tropici* CIAT 899 and derivative strains were grown at 28°C on tryptone yeast (TY) medium [57], B^−^ minimal medium [43] or yeast-extract mannitol (YM) medium [58], supplemented when necessary with apigenin to a final concentration of 3.7 μM or with NaCl at 300 mM. *Escherichia coli* strains were cultured on Luria-Bertani (LB) medium [59] at 37°C. When required, the media were supplemented with the appropriate antibiotics as described by Lamrabet *et al.* [60]. *R. tropici* RSP82 [30] was used as a *nodD1* mutant derivative of *R. tropici* CIAT899 (Km^R^ 30 μg mL^−1^).

To obtain the *nodD2* mutant, primer pairs nodD2-F (5′ – GTA GGC CAT AAT GTC CAG A) and nodD2-R (5′ – GCG GCT TTA TAC TCA CCA) were used for amplifying the *nodD2* gene. The 1450-bp PCR product was cloned into pGEM®-T Easy (Promega) (Amp^R^ 100 μg mL^−1^). The PCR-amplified *nodD2* fragment was then excised from the plasmid obtained with the endonuclease *EcoRI* and cloned into the vector pK18mob [61], which is suicide in rhizobia, confers resistance to kanamycin (km^R^ 30 μg mL^−1^) and was previously digested also with *EcoRI*. This new plasmid was digested with the enzyme *Pst*I, which cuts the *nodD2* gene in two sites, releasing a fragment of approximately 600 pb. The rest of the plasmid was treated with the Klenow enzyme to convert the cohesive ends to blunt ends. This treated plasmid was ligated with a 2-Kb DNA fragment containing the Ω interposon [carrying the spectinomycin resistance gene (spc^R^ 100 μg mL^−1^)], which was obtained from a previous digestion of the pHP45Ω plasmid [62] with the *Sma*I enzyme (blunt end). The resulting plasmid was transformed into the *E. coli* strain DH5α. Plasmids were transferred from *E. coli* to *Rhizobium* strains by conjugation, as described by Simon [63], using plasmid pRK2013 [64] as helper. The plasmid generated was used for the homogenization of the mutated version of the *nodD2* gene in *R. tropici* CIAT 899 by using the methodology previously described [65]. The homogenization was confirmed by DNA-DNA hybridization. For this purpose, DNA was blotted to Hybond-N nylon membranes (Amersham, UK), and the DigDNA method of Roche (Switzerland) was employed according to the manufacturer’s instructions. Additional file 1: Figure S1 displays the type of mutation realized to obtain the *nodD2* mutant.

The parental and mutant strains are deposited in the culture collection of the Department of Biology of the Universidad de Sevilla and at the Diazotrophic and Plant Growth Promoting Bacteria Culture Collection of Embrapa Soja (WFCC Collection # 1213, WDCC Collection # 1054).

### Identification of Nod factors

Purification and LC-MS/MS analyses of Nod factors produced by *R. tropici* CIAT 899 and derivative strains grown in B^−^ minimal medium [43] (supplemented when required with NaCl 300 mM or apigenin 3.7 μM) were performed as described previously [30].

### RNA isolation, cDNA synthesis and quantitative RT-PCR

Wild type CIAT 899 and *nodD1* and *nodD2* mutants were pre-cultured in 10-mL aliquots of TY medium at 100 rpm and 28°C in the dark. After 48 h, the three strains pre-inoculated were transferred to new media and subjected to the following conditions: control (without induction), 300 mM NaCl and apigenin 3.7 μM. These new cultures were performed in triplicate under the same conditions as for the pre-cultures, 100 rpm and 28°C in the dark, except that were grown into the exponential phase (O.D. at 600 nm of 0.5 to 0.6).

Total RNA was extracted using Trizol® reagent (Life Technologies) as previously described [66]. The total concentrations were estimated in a NanoDrop ND 1000 spectrophotometer (NanoDrop-Technologies, Inc., City etc. here and elsewhere) and the integrity was assessed by gel electrophoresis. Extracted RNA samples were submitted to DNAseI treatment (Invitrogen/Life Technologies, Grand Island, NY, USA) and the first stand of cDNA was synthesized using SuperscriptIII™ reverse transcriptase (Invitrogen™), according to manufacturer’s protocol.

Primers for the RT-qPCR targets, genes *nodD1*, *nodD2* and *nodC*, were designed using Primer3Plus (http://www.bioinformatics.nl/cgi-bin/primer3plus/primer3plus.cgi/), to obtain amplicons of 50–150 bp. With the same software, a primer to 16S rRNA was obtained and applied to normalize the relative expression of the targets. To avoid unspecific alignments, the primer sequences were searched against the *R. tropici* CIAT 899 genome (http://www.ncbi.nlm.nih.gov/nuccore/440224888?report=genbank). The primer sequences and sizes of the amplified fragments are available in Additional file 4: Table S1.

RT-qPCR reactions were performed in a 7500 RT-qPCR Thermocycler (Applied Biosystems/Life Technologies). The reactions were performed in triplicate for each of the three biological replicates. The Platinum® SYBR Green® Master Mix kit (Applied Biosystems) was used according to the manufacturer’s instructions. Cycling conditions were as follows: 50°C for 2 min, 95°C for 10 min, 45 cycles at 95°C for 2 min, 60°C for 30 s and 72°C for 30 s, in 45 cycles. Rest2009 software package [67] was used to evaluate the data by providing a robust statistical analysis (*p* < 0.05). The normalization of cycle threshold (Ct) of RT-qPCR amplifications was performed based on the selected endogenous gene (16S rRNA).

### Studies of external exopolysaccharides

The anthrone-H_2_SO_4_ method, which measures the total reducing sugar content in a given sample [68] was used to determine the total carbohydrate amounts of exopolysaccharide (EPS) contained in supernatants from bacterial cultures. For this purpose, *R. tropici* CIAT 899 and derivatives were grown in 5 mL of TY liquid medium on an orbital shaker (180 rpm) for 96 h at 28°C. When required, the media were supplemented with NaCl (300 mM) or apigenin (3.7 μM). Samples of 1 mL were centrifuged to remove cells. Cell-free culture supernatants were assayed for EPS content via H_2_SO_4_ hydrolysis in the presence of the colorimetric indicator anthrone. Every experiment was performed three times with three replicates each time. Lipopolysaccharide (LPS) extraction, separation on SDS-PAGE, and silver staining were performed as previously described using the same bacteria, medium and conditions [17].

### Motility assays

Swimming and swarming phenotypes were tested on TY medium [57] (supplemented when necessary with NaCl 300 mM or apigenin 3.7 μM) agar plates containing 0.28% or 0.4%, respectively, of Bacto Agar. The strains to be assayed (wild type and mutants) were grown in 5 mL of TY medium on an orbital shaker (180 rpm) for 96 h at 28°C. Aliquots (2 μL) of culture suspensions were drop-inoculated (swarming assay) or sink-inoculated (swimming assay) onto plates and air-dried in a laminar-flow cabinet. The inoculated plates were wrapped with parafilm and incubated for the required time at 28°C in an upright position. Every experiment was performed three times with three replicates each time.

### Biofilm formation assay

The biofilm formation assay on polystyrene surfaces was performed using the method described by O’Toole and Kolter [69] with modifications [20]. CIAT 899 and mutant strains were grown on TY medium [57] (supplemented with NaCl 300 mM or apigenin 3.7 μM when required) for 7 days with gentle rocking at 28°C. Every experiment was performed three times with eight replicates each time.

### Quantification of indole acetic acid (IAA) production

Quantification of an IAA-like compound from *R. tropici* strain cultures was carried out by using Salkowski colorimetric assays [70], as described previously by Fierro-Coronado *et al.* [71]. To measure IAA production, 5 mL of TY medium with tryptophan (0.4 g L^−1^) (supplemented when required with NaCl 300 mM or apigenin 3.7 μM) were inoculated and incubated during 96 h at 28°C on an orbital shaker (180 rpm) with *R. tropici* strains. Of these cultures, samples of 1 mL were centrifuged to remove cells. Cell-free culture supernatants were assayed for IAA production. Every experiment was performed three times with eight replicates each time.

### Nodulation assays

*nodD1* and *nodD2* mutants were grown in YM medium until a concentration of 10^9^ cells mL^−1^ was achieved, to be used as inoculum. Surface-sterilized seeds [58] were used for the assays with common bean (*Phaseolus vulgaris* L.), leucaena [*Leucaena leucocephala* (Lam.) de Wit] and siratro [*Macroptilium atropurpureum* (DC) Urb.]. Pre-germinated seeds (about 2 days after germination) were placed in sterilized pouches or Leonard jars containing N-free nutrient solution [58], with 1 mL of inoculum of each strain added and verified for nodulation capacity after 25 (common bean) or 42 days (leucaena and siratro) with a 16-h 25C°/18°C photoperiod and about 70% relative humidity. Shoots were dried at 65°C until constant weight was achieved, and then weighed. Experiments were performed three times.

## Additional files

Additional file 1: Figure S1. Information about the *nodD1* and *nodD2* genes of *R. tropici* used in our study. A. Gene neighborhood of *nodD1* and *nodD2* genes in the genome of *R. tropici* strain CIAT 899. B. Location of primers (dark arrows) used to perform RTqPCR experiments. C. Schematic representation of the *nodD2* mutation. Additional file 2: Figure S2. Nodulation phenotype in common bean (*Phaseolus vulgaris*) inoculated with CIAT 899 and derivative *nodD* strains assayed in pouch bags. Experiment performed under controlled conditions of growth chamber and plants harvested at 25 days after inoculation. A. wild type strain. B. *nodD1* mutant. C. *nodD2* mutant. D. Uninoculated. Additional file 3: Figure S3. Main properties observed in wild type (WT), *nodD1* and *nodD2* mutants of *R. tropici* strain CIAT 899. Additional file 4: Table S1. Sequences of the primers used in the RT-*q*PCR and sizes of the PCR products obtained.
