# Supplementary figures and images for: Regulatory nodD1 and nodD2 genes of Rhizobium tropici strain CIAT 899 and their roles in the early stages of molecular signaling and host-legume nodulation
Source: BMC Genomics. 2015 Mar 28;16(1):251. doi: 10.1186/s12864-015-1458-8 (PMC4393855; doi:10.1186/s12864-015-1458-8)

**Figure S1**


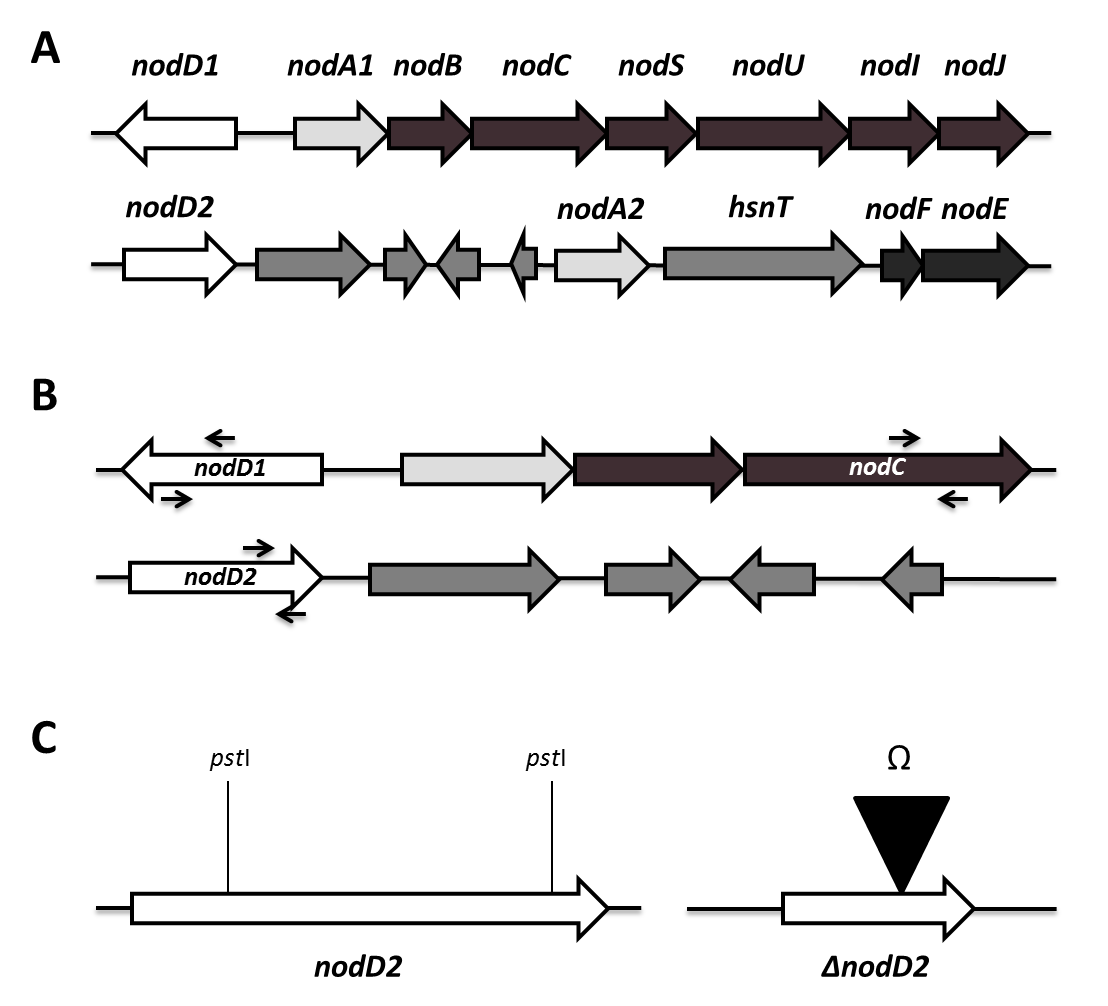

Supplement: Additional file 1: Figure S1. — Information about the nodD1 and nodD2 genes of R. tropici used in our study. A. Gene neighborhood of nodD1 and nodD2 genes in the genome of R. tropici strain CIAT 899. B. Location of primers (dark arrows) used to perform RTqPCR experiments. C. Schematic representation of the nodD2 mutation. [file 12864_2015_1458_MOESM1_ESM.docx]

**Figure S2**


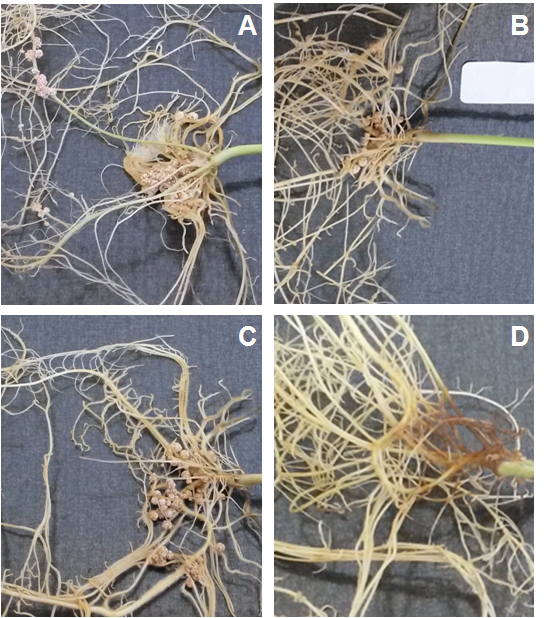

Supplement: Additional file 2: Figure S2. — Nodulation phenotype in common bean (Phaseolus vulgaris) inoculated with CIAT 899 and derivative nodD strains assayed in pouch bags. Experiment performed under controlled conditions of growth chamber and plants harvested at 25 days after inoculation. A. wild type strain. B. nodD1 mutant. C. nodD2 mutant. D. Uninoculated. [file 12864_2015_1458_MOESM2_ESM.docx]
