# Supplementary material for: Regulatory nodD1 and nodD2 genes of Rhizobium tropici strain CIAT 899 and their roles in the early stages of molecular signaling and host-legume nodulation
Source: BMC Genomics. 2015 Mar 28;16(1):251. doi: 10.1186/s12864-015-1458-8 (PMC4393855; doi:10.1186/s12864-015-1458-8)
Supplement: Additional file 3: Figure S3. — Main properties observed in wild type (WT), nodD1 and nodD2 mutants of R. tropici strain CIAT 899. [file 12864_2015_1458_MOESM3_ESM.pptx]

## Slide 1
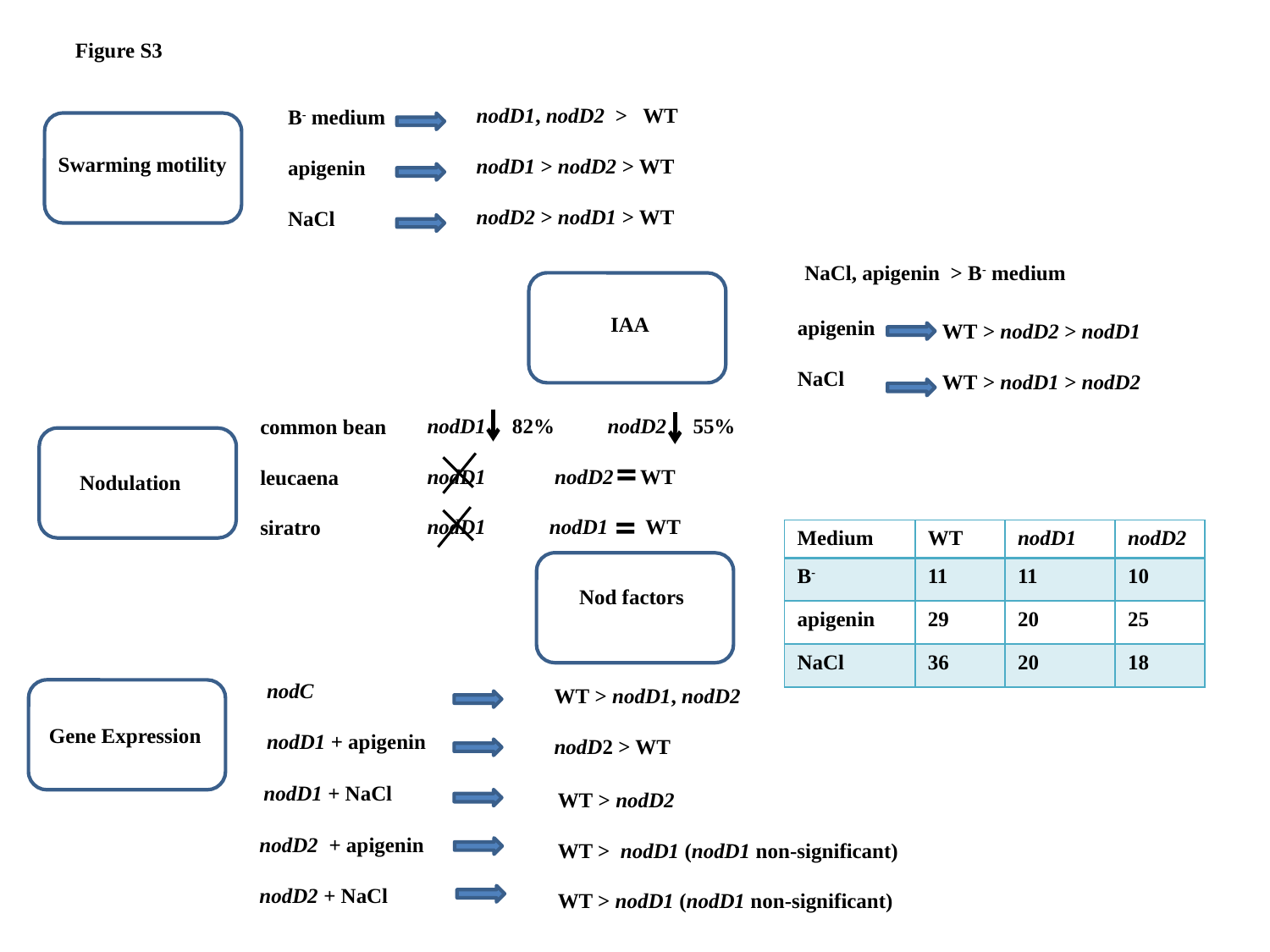

Figure S3
nodD1, nodD2 > WT
nodD1 > nodD2 > WT
nodD2 > nodD1 > WT
B- medium
apigenin
NaCl
Swarming motility
NaCl, apigenin > B- medium
IAA
apigenin
NaCl
WT > nodD2 > nodD1
WT > nodD1 > nodD2
nodD1 82% nodD2 55%
nodD1 nodD2 WT
nodD1 nodD1 WT
common bean
leucaena
siratro
=
Nodulation
=
| Medium | WT | nodD1 | nodD2 |
| --- | --- | --- | --- |
| B- | 11 | 11 | 10 |
| apigenin | 29 | 20 | 25 |
| NaCl | 36 | 20 | 18 |
Nod factors
nodC
nodD1 + apigenin
WT > nodD1, nodD2
nodD2 > WT
Gene Expression
nodD1 + NaCl
WT > nodD2
WT > nodD1 (nodD1 non-significant)
WT > nodD1 (nodD1 non-significant)
nodD2 + apigenin
nodD2 + NaCl
