# Supplementary material for: Regulatory nodD1 and nodD2 genes of Rhizobium tropici strain CIAT 899 and their roles in the early stages of molecular signaling and host-legume nodulation
Source: BMC Genomics. 2015 Mar 28;16(1):251. doi: 10.1186/s12864-015-1458-8 (PMC4393855; doi:10.1186/s12864-015-1458-8)
Supplement: Additional file 4: Table S1. — Sequences of the primers used in the RT-qPCR and sizes of the PCR products obtained. [file 12864_2015_1458_MOESM4_ESM.docx]

**Table S1 Sequences of the primers used in the RT-*q*PCR and sizes of the PCR products obtained.**

| **Name** | **Sequence** | **Amplicon** |
| --- | --- | --- |
| *nodD1* F | 5'- AAGAGCTATCACAGCCGCTTAC -3´ | 116 pb |
| *nodD1* R | 5'- ACCGTGTTCGAGCAAATACC -3´ |  |
| *nodD2* F | 5'- GAATTGTGGAAAGTCCAATA 3´ | 116 pb |
| *nodD2* R | 5'- CGCCTCCTGCAGAATTATCT -3´ |  |
| *nodC* F | 5'- CAAGCTGCGCCCTTATCTG -3´ | 127 pb |
| *nodC* R | 5'- CAAGCAACGTGTCACGGAAA -3´ |  |
| 16S rRNA F | 5'- ACACACGTGCTACAATGGTG -3´ | 129 pb |
| 16S rRNA R | 5'- GCGATTACTAGCGATTCCAA -3´ |  |
